# How do stroke early supported discharge services achieve intensive and responsive service provision? Findings from a realist evaluation study (WISE)

**DOI:** 10.1186/s12913-023-09290-1

**Published:** 2023-03-28

**Authors:** Niki Chouliara, Trudi Cameron, Adrian Byrne, Sarah Lewis, Peter Langhorne, Thompson Robinson, Justin Waring, Marion Walker, Rebecca Fisher

**Affiliations:** 1 NIHR Applied Research Collaboration (ARC) East Midlands, School of Medicine, University of Nottingham, Nottingham, England; 2 School of Medicine, University of Nottingham, Nottingham, England; 3 School of Cardiovascular & Metabolic Health, University of Glasgow, Glasgow, Scotland; 4 Department of Cardiovascular Sciences, University of Leicester, Leicester, England; 5 Health Services Management Centre, University of Birmingham, Birmingham, England

**Keywords:** Early supported discharge, Stroke, Community-based stroke rehabilitation, Evidence-based practice, Realist evaluation, Rehabilitation intensity, Service responsiveness, Qualitative interviews

## Abstract

**Background:**

Stroke Early Supported Discharge (ESD) involves provision of responsive and intensive rehabilitation to stroke survivors at home and it is recommended as part of the stroke care pathway. Core components have been identified to guide the delivery of evidence-based ESD, however, service provision in England is of variable quality. The study sought to understand how and in what conditions the adoption of these components drives the delivery of responsive and intensive ESD services in real world settings.

**Methods:**

This qualitative study was part of a wider multimethod realist evaluation project (WISE) conducted to inform large-scale ESD implementation. Overarching programme theories and related context–mechanism–outcome configurations were used as a framework to guide data collection and analysis. Six case study sites were purposively selected; interviews and focus groups with ESD staff members were conducted and analysed iteratively.

**Results:**

We interviewed 117 ESD staff members including clinicians and service managers. Staff highlighted the role of certain core components including eligibility criteria, capacity, team composition and multidisciplinary team (MDT) coordination in achieving responsive and intensive ESD. Regardless of the geographical setting, adhering to evidence-based selection criteria, promoting an interdisciplinary skillset and supporting the role of rehabilitation assistants, allowed teams to manage capacity issues and maximise therapy time. Gaps in the stroke care pathway, however, meant that teams had to problem solve beyond their remit to cater for the complex needs of patients with severe disabilities. Adjusting MDT structures and processes was seen as key in addressing challenges posed by travel times and rural geography.

**Conclusions:**

Despite variations in the wider service model of operation and geographical location, the adoption of core components of ESD helped teams manage the pressures and deliver services that met evidence-based standards. Findings point to a well-recognised gap in service provision in England for stroke survivors who do not meet the ESD criteria and emphasise the need for a more integrated and comprehensive stroke service provision. Transferable lessons could be drawn to inform improvement interventions aimed at promoting evidence-based service delivery in different settings.

**Trial registration:**

ISRCTN: 15,568,163, registration date: 26 October 2018.

Contribution to the literature

- Findings highlight the value of distinguishing between evidence-based intervention components that need to be safeguarded and elements that can be tailored to local context.
- Adherence to evidence-based selection criteria, the composition and co-ordination of the multidisciplinary team, and the availability of other community rehabilitation services in the stroke care pathway, may influence the ability of ESD services to provide an intensive and responsive service.
- Rather than being studied in isolation, the realist evaluation approach allowed us to examine the influence of context as integral to the process generating the outcomes of interest.

## Background

Stroke is one of the largest causes of adult disability with lasting impact on people’s lives and healthcare systems [1]. Research evidence has demonstrated the benefits of stroke specialist rehabilitation in promoting recovery [2]. In England, the National Health Service (NHS) Long Term Plan has highlighted the need for improved post hospital rehabilitation service models [3]. The challenge of cost reduction and integrated care provision further stresses the need for effective home-based stroke rehabilitation care [3, 4].

Early Supported Discharge (ESD) involves the co-ordinated transfer of care from hospital to the patient’s home to provide timely and specialist rehabilitation by a multidisciplinary team (MDT) [5]. Driven by a strong evidence-base on the benefits of the intervention, stroke care guidelines, in England and worldwide, recommend the provision of ESD as part of an evidence-based stroke care pathway [2, 6–9]. To promote patient recovery, research and clinical guidelines recommend that ESD services should be responsive, with treatment at home beginning within 24 h of hospital discharge and at an intensity similar to what patients would receive in hospital [2, 3, 10]. Although the importance of these aspects of an effective ESD has been demonstrated in clinical trials, it remains unclear whether and how the delivery of responsive and intensive service is achieved in routine clinical practice.

With the majority of national audits or registries focusing on acute stroke care, there is currently insufficient evidence on the large-scale implementation of ESD in real world settings. In England, the Sentinel Stroke National Audit Programme (SSNAP) showed that, where available, the provision of ESD is variable and can differ from evidence-based models [11, 12]. It has, therefore, been unclear whether the benefits demonstrated in clinical trial settings are achieved when ESD services are implemented in practice. Seen against the backdrop of a complex healthcare landscape, the observed variability may reflect differences in service resourcing, workforce planning or attempts to address local needs. Understanding how context influences and shapes ESD implementation is necessary to address inequities in service provision and inform service improvements.

It has been suggested that complex interventions could be conceptualised as consisting of core active ingredients and an adaptable periphery that can be modified to account for contextual influences [13]. Our previous work has identified core components of ESD interventions, based on an international ESD consensus document and evidence-based standards [2, 10]. Among others, they include the delivery of a stroke specialist service by a co-ordinated MDT, to eligible patients in their own home. Adoption of these components is expected to result in delivery of effective services in practice, as indicated by responsive and intensive service delivery [14].

What is less understood is how these core components are achieved in real world settings and how they interact with contextual features to lead to the outcomes of interest. The experiences of clinicians responsible for delivering ESD interventions on the ground is key to investigating adherence to evidence-based standards and the delivery of effective services in practice.

This qualitative study was conducted under a wider multimethod project (i.e. What Is The Impact of Stroke ESD; WISE) designed to inform large scale implementation of ESD [15]. The aim of this study was to examine how and in what circumstances adopting core components drives the delivery of intensive and responsive ESD services, from the perspective of staff members.

## Methods

### Methodological framework

This study adopted qualitative research methods under a realist evaluation methodological approach [16] and it was part of a larger multimethod research project (i.e. What Is The Impact of Stroke ESD; WISE) assessing ESD implementation in real world settings [15]. Realist Evaluation (RE) is a widely used methodology with established quality criteria [17]; it seeks to develop, test and refine theories that explain how healthcare interventions work and why they are successful in certain conditions and not in others. The UK Medical Research Council’s guidance for complex interventions highlights the need to theorise how and under what conditions complex healthcare programmes bring about change [18]. To develop and refine “programme theories” and support real world decision making, the framework recommends drawing on diverse stakeholder perspectives. Our realist methodological approach responds to these priorities for complex intervention research to successfully address the study’s aims.

Programme theories in RE consist of Context-Mechanisms-Outcomes (CMO) configurations which formulate hypotheses about how contextual factors interact with underlying mechanisms to generate intended or unintended outcomes [17]. Mechanisms could be defined as an interaction between the resources offered by the intervention and stakeholders’ reasoning and responses to these resources.^19^As part of the larger study, we developed three preliminary programme theories, informed by our previous research and national and policy guidelines on stroke ESD [2, 10, 19, 20]. These a priori theories guided the development and testing of CMOs through data collection and analyses. The first theory tested the assumption that evidence-based core components, as identified by research and national clinical guidelines [2, 10], are essential characteristics that need to be implemented for ESD intervention to be effective in practice. These core components include that ESD is delivered by stroke-specialist staff operating as a co-ordinated multidisciplinary team [e.g. physician, physiotherapist, occupational therapist (OT), and speech and language therapist (SLT)]; that the ESD team has weekly MDT meetings and regular meetings with stroke unit hospital staff; and that an effective ESD intervention consists of co-ordinated, facilitated discharge from hospital and the provision of timely and intensive rehabilitation for eligible (mild to moderate) stroke patients at home [10].

The formal theory implied by the evidence base would suggest that in urban settings (Context), the provision of timely hospital discharge and intensive home rehabilitation (Mechanism) by co-ordinated, stroke specialist multidisciplinary ESD teams (Mechanism), reduces length of in-hospital stay and improves long-term functional outcomes (Outcomes).However, this theory does not explain how these components operate in contexts other than urban environments and how they are influenced by known and unknown contextual determinants. It also assumes a causal relationship between the intervention characteristics and the outcomes, failing to consider the effect of human agency, the perspectives and behaviours of those involved with the implementation of the intervention [21]. Framed in realist terms, the first programme theory tested in this study conceptualised the evidence-based core components of ESD services as the programme resources, and the stakeholders’ (staff and patients) reasoning and responses to these resources as the underlying mechanisms that interact with the context to produce intended and unintended outcomes (Table 1).

**Table 1:** Programme theory framework

Contex	Mechanisms		Outcomes
	**Resources** ^a^	**Responses**	
Service capacity Geographical location Commissioning and financial arrangements ESD Provider organisation Referring services characteristics & locations	Eligibility criteria Team composition Whole time equivalent Staff/patient ratio Stroke specialism & staff training Multidisciplinary team co-ordination (e.g. meetings)	Staff perspectives/ behaviour Patient perspectives/ behaviour	Rehabilitation delivery – responsiveness Rehabilitation delivery – intensity of rehabilitation

Example of a CMO configuration with intended outcomes: “If services covering rural areas have telerehabilitation technologies in place, then MDT members may use flexible working arrangements effectively, helping to maximise resources and optimise service responsiveness and intensity.”

Example of a CMO configuration with unintended outcomes: “If ESD services covering rural geographical areas offer on-site rehabilitation groups to augment intensity, but support with transport is not available, patients living remotely might refuse attendance and receive less therapy that patients living closer to the service.”

^a^Core components of evidence-based ESD services

The second programme theory suggested that core components of ESD will operate differently in urban and rural settings. With most trials conducted in urban settings, the delivery of ESD services in dispersed rural communities needs to be assessed. The influence of the geographical location within which services operate was therefore a key contextual factor we wanted to investigate and the focus of the second programme theory. A third programme theory was developed to explore the influence of the quality of communications between stakeholders, but it is presented elsewhere [22].

The framework presented in Table 1 underpinned the development of preliminary Context-Mechanisms-Outcomes (CMOs) configurations that were assessed and refined through data collection and analysis. Our exploration of context was informed by a Rapid Evidence Synthesis (RES) [23] ,which preceded this study. The RES process involves summarising and synthesising evidence from quantitative and/or qualitative studies to address specific research questions. Our aim was to identify key contextual determinants, facilitating or impeding the implementation of home-based stroke rehabilitation, which we should consider in developing our CMO hypotheses [22].

Regarding the outcomes, this study focused on the responsiveness (number of days from hospital discharge to first face-to-face contact) and intensity of ESD service provision, which are key process measures of ESD effectiveness and are routinely assessed as part of SSNAP. These outcomes were also investigated quantitatively in a parallel study [14]. Table 1 presents examples of how the theoretical framework informed CMO development.

### Sampling and data collection

As part of the wider project, a realist sampling frame was developed to purposively select study sites and participants with characteristics that mattered within our programme theories [24]. Six ESD services in England were selected to capture variation in relation to: (1) geographical regions in England, (2) models of ESD provision and (3) rurality levels [25]. Information to characterise the different models of care included the size of the team (i.e. patient caseload and number of staff) and the adoption of core components including team composition, staff training, team meetings and eligibility criteria.

Purposive and snowball strategies were used to identify key informants from each of the participating sites. One-to-one interviews were conducted with service leads, managers and commissioners at each ESD site [26]. We also conducted two group interview sessions at each site with a cross-section of the multidisciplinary team (MDT), including physicians, therapists, nurses, rehabilitation assistants, and administrators [27].

All one-to-one interviews were conducted by NC (1st author, PhD) and focus groups were conducted by both NC and TC (2nd author, PhD). NC and TC were research fellows on the study with significant experience in conducting interviews and focus groups on stroke rehabilitation topics. Potential participants were first contacted over the phone/email, but all data collection was conducted face-to-face.

Prior to the interviews, researchers introduced themselves, presented the study’s scope and aims, explained how the data would be used, and obtained written informed consent. Semi-structured interview schedules reflecting a realist interview approach were developed and pilot tested with 5 clinicians not involved in the study.

Participants were invited to reflect on preliminary CMO configurations (informed by Table 1) and, in doing so, share their perspectives on contextual conditions and processes that influence their service model and effectiveness. This process allowed researchers to directly test their assumptions and hypotheses with participants. Emerging themes and issues informed subsequent interviews, [28] contributing to the richness and robustness of the data collected. Recruitment at each site was completed when the sampling aims were achieved i.e. the views of a cross-section of stakeholders were captured. Interviews were conducted at each ESD site, between September 2018 and August 2019, and lasted between 45 min and 2 h (for group interviews). Audio recordings were transcribed verbatim and imported into QSR International NVivo 12 Pro for Windows.

### Data analysis

In line with a realist approach, data were analysed following an iterative, retroductive process moving between deductive and inductive phases [28]. Starting with a programme’s outcomes, retroductive analysis involves working backwards to identify contextual determinants and generative mechanisms and theorise how their interaction leads to the outcomes of interest [29].

Our candidate programme theories and preliminary CMO configurations formed an initial framework used to guide the analysis and develop a codebook (deductive approach) [30] Participants’ narratives were examined to identify potential links between contexts, mechanisms and outcomes and code them into C-M-O strings.^32^During the coding process, however, we also looked for examples that confirmed or disconfirmed our framework, allowing for revisions and unexpected findings to be identified (inductive approach). As the analysis went on, the framework was developed and refined in response to new insights.

The coding process involved two key stages:

1. Participant-level data.

At this stage, the units of analysis were individual interviews and focus groups. The same coding and analysis processes were followed for both of these types of interview. Service descriptions synthesised from interviews and documentary evidence provided background information on the operation of each service. Analysis started by reading through each transcript to map out where different topics and theories were being introduced and identify C-M-Os. Where connections between C-M-O elements could be discerned from participants’ narratives, these were coded as either triads (where all three elements were present) or dyads (e.g. C–M, M–O combinations) [31]. CMO strings and associated text extracts were coded under the relevant programme theories but they could be revisited/refined at a later stage of analysis as the connections C-M-Os became better understood. Where it was not possible to identify dyads or triads but the inclusion of a text extract was considered important, the extract was coded as referring to distinct C-M-O processes.

2. Site-level data

Once the analysis of interviews from each site was completed, cross-site comparisons were conducted to identify confirming and disconfirming cases and explore how pertinent mechanisms interact with site-specific contextual conditions to generate variation in outcomes. The identified CMO configurations were related back to the original programme theories and further refinements were made.

The analysis was conducted by two members of the research team (NC and TC). They initially coded data independently, producing tables of CMO configurations related to each overarching programme theory. They then discussed their analysis and insights with the rest of the research group until agreement was reached that no further revisions and refinements were required.

## Results

As shown in Table 2 in total we spoke to 117 staff members. No respondents withdrew participation at any point. In the results section we present key CMO configurations, organised thematically under relevant headings based on common mechanisms. These mechanisms related to core components included in our theoretical framework presented in Table 1 (i.e. eligibility, staff/patient ratio, team composition, MDT coordination). To facilitate the reader, descriptions corresponding to Context, Mechanisms and Outcome elements are indicated in the text in brackets. The narrative is complemented by illustrative quotes and figures showing how the interaction between identified contexts and mechanisms led to intended (in green) or unintended outcomes (pink boxes).

**Table 2:** Numbers of staff participants per site

Site^a^	Individual interviews (n)	Focus group attendees (n)	Total staff participants (n)
**A**	5	12	17
**B**	5	15	20
**C**	8	9	17
**D**	2	13	15
**E**	9	16	25
**F**	6	17	23
**Total**	**35**	**82**	**117**

^a^A and B sites offer both ESD and community rehabilitation; Sites A and B covered an urban location, sites C and D a semi-rural location and sites E and F a rural location

### Accessing the service: eligibility (Fig. 1)

**Fig. 1:**
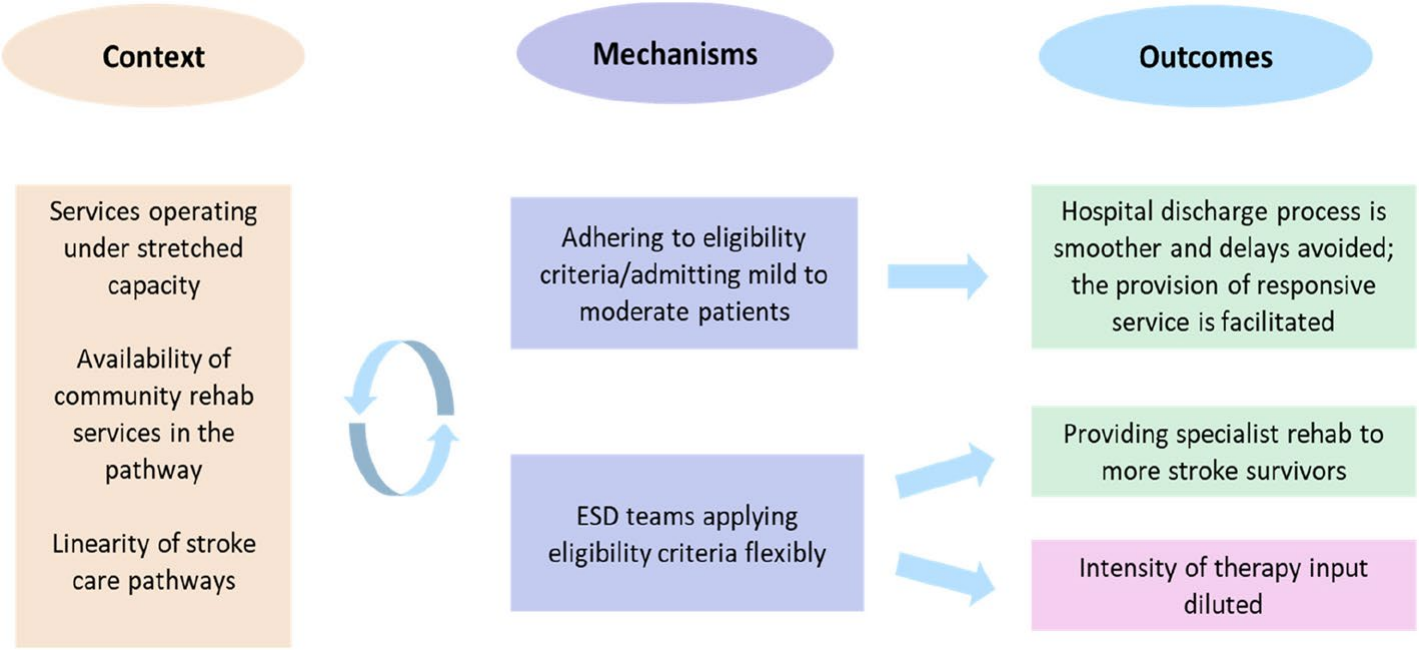
The CMOs on eligibility

#### Adhering to eligibility criteria

ESD services adhered to eligibility criteria to manage access to the service, with a focus on providing ESD to stroke survivors with mild to moderate disability, based on evidence and guidelines [2, 10]. Against a background of capacity issues (C) adherence to these eligibility criteria (M), promoted the provision of intensive and responsive ESD (O). Reasons included reducing the complexity of the hospital discharge process for people with severe disabilities, such as ensuring safety at home, avoiding delays related to securing social care packages and maximising capacity.

*“We are very strict with eligibility. it’s supposed to be a virtual ward and it should be an intense service. We are able to provide that intense service by not offering too many slots depending on how many staff you have”. (*Site C, semi-rural, *focus group)*

*“If we had more severe strokes, we’d need at least two members of staff every single time and it would just take more people and more handling and we haven’t got the capacity.” (Site E, rural, focus group)*.

Adherence to eligibility criteria was influenced by the availability and quality of follow-up community rehabilitation services in the catchment area (C). For patients who were not eligible for ESD, it could be months before they were admitted to a community rehabilitation service or they could be discharged without any further rehabilitation (C). Patients who were deemed unable to manage the intensity of ESD (e.g. due to fatigue or infection) at the point of hospital discharge were not offered further opportunities for specialist rehabilitation (C).

*“The difficulty at the moment is you might have someone who, in the early stages, takes a long time to rehabilitate but say in three months’ time they start to make some progress, but unfortunately they’re then out of the stroke pathway.”(Site A, urban, clinical team lead)*.

In this context, it was common for ESD services and/or referrers to either stretch the eligibility criteria or delay hospital discharge (M). In light of capacity issues, however, attempting to cater for the complex needs of patients with more severe disabilities could dilute rehabilitation intensity for the rest of the patients (O). It was suggested that a more integrated stroke care pathway would be required if ESD services were to effectively support these patients at home.

*“There would have to be very clear integrated working. That’s not just a decision that can be made by an ESD team providing it. It’s also about whether ambulance would support it, whether social services would support that, whether the carers want it”. (Site E, rural, service manager)*

#### Staff/patient ratios (capacity) (Fig. 2)

**Fig. 2:**
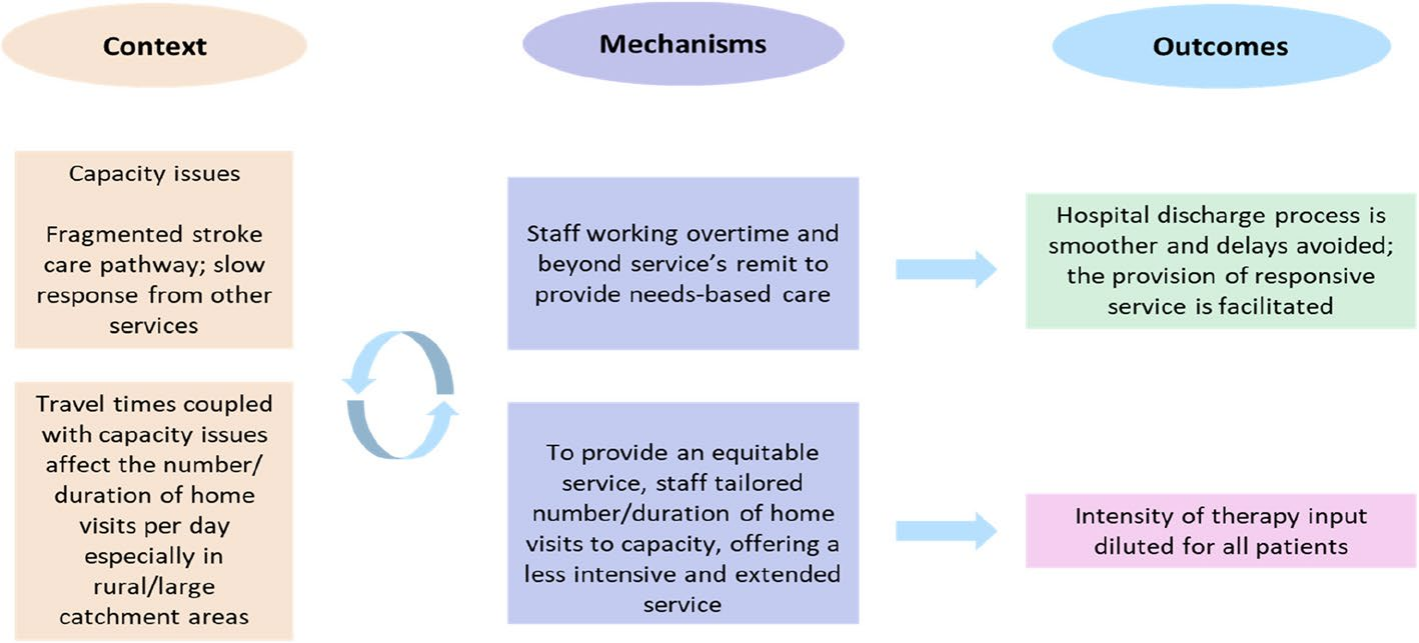
The CMOs on capacity

Services reportedly struggled to manage the tension between responding to an expanding list of referrals and offering the intensity required to achieve improvements in patient outcomes (C). Teams often relied on staff “walking the extra mile” and working longer hours (M) to keep up with demand and meet intensity targets (O). One team had started documenting unmet visits to draw commissioners’ attention to the problem and evidence the need for more staff.

*“We’ve been over our capacity for a while now and commissioners think we are managing (…) We have started documenting when we can’t see patients. It’s hard accepting that you can’t always meet the targets, but we have to not meet them in order to get that evidence to show that we can’t. I think that’s the trouble. Because we’re such a good team and we are conscientious, we will just do our best to meet all these targets”. (Site D, semi-rural, focus group)*

Gaps in service provision and slow response from other services, including GPs and social care(C), often meant that ESD teams had to problem solve beyond their remit to provide needs-based care (M). Therapists could be called to address unmet information needs or liaise with other services to ensure patients received the correct medication and support they required. Faced with capacity issues, their efforts to help patients navigate a fragmented stroke care pathway detracted from hands-on rehabilitation. Though they could be spending more than the recommended 45 min with their patients, the intensity of active rehabilitation was diluted (O).

*“We often become problem solvers for the wider picture. We’re also the end of the line. The acute unit can be under a lot of pressure to discharge and mistakes and omissions will inevitably happen. Once that person is discharged, whoever is at the end of the line has a duty of care and has to pick up any loose ends. You end up being more of a case worker or a kind of key worker which can take up a huge amount of time. If you’re taking on the kind of active role of trying to signpost them on to other places, that can detract from any kind of therapeutic input. It’s therapeutic within itself, but the goals that we get judged on can be affected due to that.” (Site D, semi-rural, focus group)*.

Respondents identified the time required to travel to their patients (C) as an important element of context, which could influence the number of patients they could see in a day and the intensity that they could offer. The impact was more pronounced in services covering rural and/or large catchment areas (C) where teams spent a big part of their day on the road, leaving less time for active rehabilitation. To ensure that patients living remotely did not receive a lesser service, these teams spread the number of visits evenly across their patients; when capacity was stretched, however, they also had to extend the length of the service to ensure patients achieved rehabilitation goals before discharge (M). Although this strategy ensured that all patients were equally affected by capacity issues, it also meant that they received a less-intensive service over a longer period of time (O).

*“If our case load is higher, they get less visits; in which case, their length of stay is longer because they’re not getting that intensity but we try to be fair with all of our patients and share those visits out.” (Site E, rural, service manager)*.

### Team composition (Fig. 3)

**Fig. 3:**
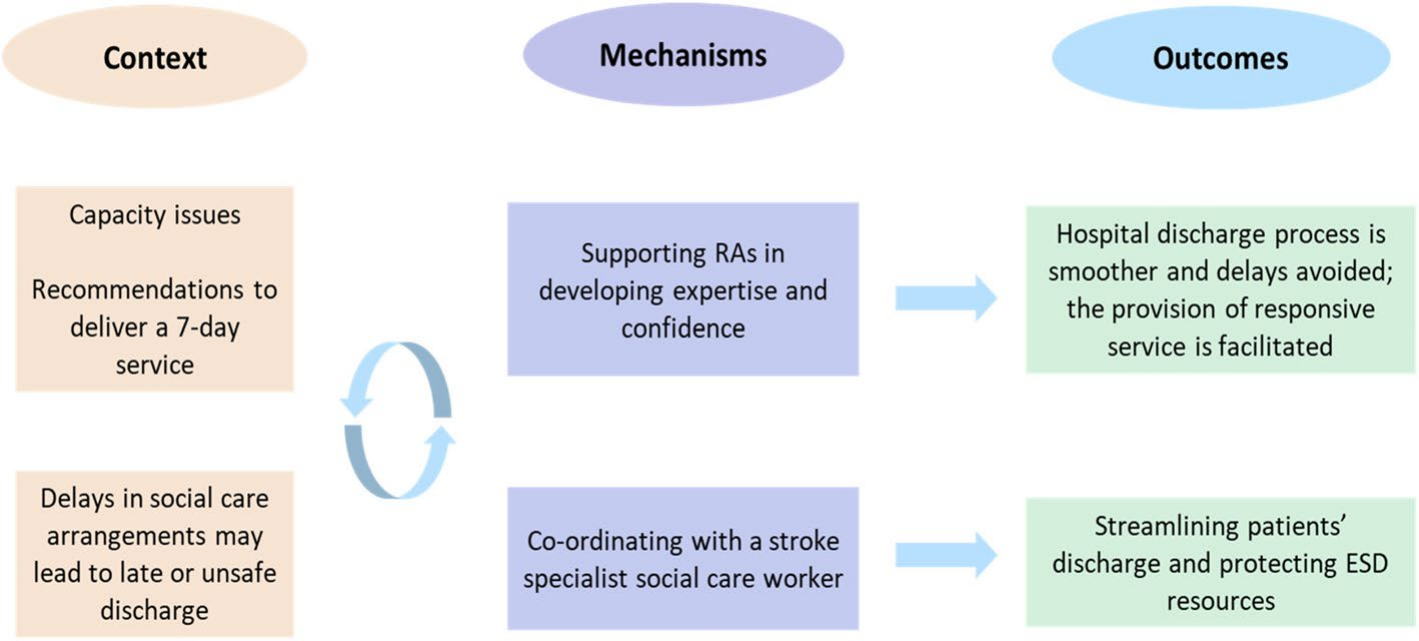
The CMOs on team composition

Respondents agreed that rehabilitation assistants (RAs) had a key role to play in the MDT. To manage capacity issues (C), teams supported RAs in developing an interdisciplinary skillset, spanning disciplinary boundaries, and building their confidence under appropriate supervision (M) to maximise therapy time (O).

*“The rehabilitation assistants will see the more high-level patients, then that reduces the load on us (therapists) and we then have the capacity to see the more complex patients.” (Site E, rural, physiotherapist)*.

In some teams, RAs were empowered to handle complex cases and escalate when necessary. Some teams trained generic rehabilitation assistants to complete initial holistic assessments and provide weekend cover; this enabled them to improve responsiveness and respond to recommendations for a 7-day service (O).

*“You need the therapy in there as quickly as possible, but we need to ensure it’s a safe discharge. (…) Some of the rehabilitation assistants, their initial assessments are far superior really, making sure they’ve covered everything”. (Site F, rural, occupational therapist)*

Delays in social care arrangements coupled with poor communication between ESD services and the referring teams could prolong hospital length of stay and lead to late or unsafe discharges (C). The capacity of ESD services was also affected, as slots were unnecessarily reserved, limiting patient flow from the hospital (C). The problem was mitigated through the involvement of a stroke specialist social worker who would follow patients and their families on their journey through the stroke pathway (M). Their work across social and health care organisational boundaries, facilitated timely information exchange, allowing the ESD service to allocate targeted admission slots and ensure patients were seen soon after their return home (O). One team contrasted between rehabilitation units with and without social workers:

*“Late notice discharges is a problem for us. Hospital [X] have their own social worker, so they’re much more precise about when their patients are discharged, and that makes it much easier for them to refer a week early, and the process is smoother.” (Site C, semi-rural, speech and language therapist)*.

### MDT coordination (Fig. 4)

**Fig. 4:**
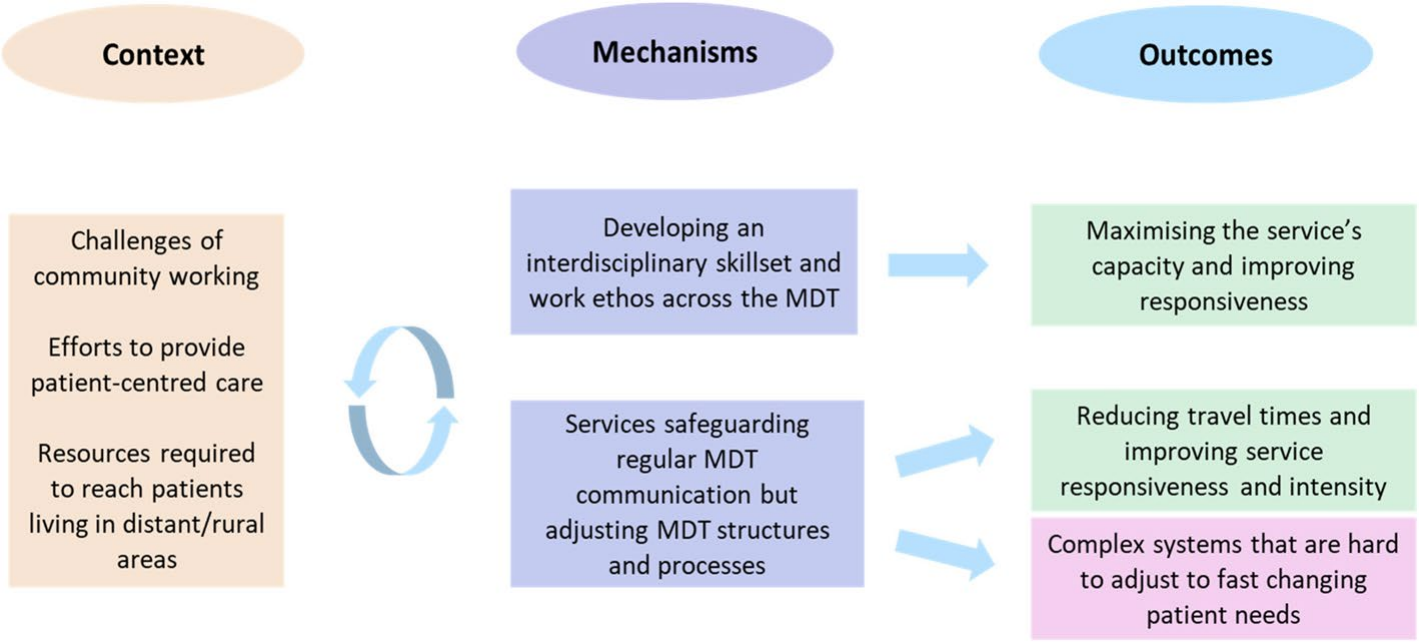
The CMOs on MDT coordination

Respondents across sites agreed that the challenges of community working (C), such as travel times, called for interdisciplinary working and blurring of professional divides (M) to provide intensive rehabilitation input in an efficient way (O). In addition to promoting teamwork and collegiality, developing an interdisciplinary skillset enabled MDT members to coordinate effectively and work flexibly across professional boundaries to best respond to patients’ needs. The term ‘nerapists’ was introduced by one team to describe their nurses’ and therapists’ shared skillset and approach to rehabilitation.

*“You have still got your unique role but there is a lot of blurring of boundaries. We work in a very interdisciplinary way so that there is that overlap of roles really. So that you don’t have to say “Well sorry that’s your upper limb, I will get the OT to come”. The physio can do things and vice versa because they understand each other’s role and they are very comfortable with that.” (Site B, urban, service manager)*.

Remote working arrangements helped to reduce travel time, but also created less opportunities for face-to-face interaction and exchange of information, expertise and emotional support between MDT members. Services covering rural and/or large geographical areas took active steps to mitigate the impact of travelling on the operation of their services and promote MDT communication and coordination (C). Whilst they safeguarded regular MDT meetings, they also adjusted MDT structures and processes (M). For instance, some teams implemented ‘patch working’ which involved dividing their MDTs into sub-teams that covered different geographical areas. Sub-teams would work flexibly to keep caseloads evenly spread and allocate resources where they were most needed, a strategy thought to maximise capacity and improve responsiveness (O).

*“We work in geographical locations so that the staff have to travel the least distances to make it as efficient as possible (…) and patients are seen the next working day of referral. There’s someone who covers the north and someone who covers the south every day so that they can get seen in a timely manner. (…) We do blend obviously if somebody’s got a big caseload”. (Site A, urban, physiotherapist)*

Sophisticated time-tabling systems were also implemented by these teams which were viewed as a way to optimise MDT planning and co-ordination. Although these systems and processes helped the teams address some of the challenges posed by geography, they also made the way they worked more rigid, allowing clinicians little flexibility to respond to patients’ fast changing needs (O). For instance, arranging short but more frequent visits to manage patients’ fatigue was a challenge for patients living remotely/rurally. On the other hand, patients who were easier to access could, reportedly, benefit from cancellations and opportunistic visits or even experience a more responsive service.

*“If there’s someone just down the road and someone’s had a cancellation, they could quickly nip out to see them where they’ve got a slot, whereas if they’re right at the top of north of the county then they can’t”. (Site E, rural, focus group)*

## Discussion

This study examined how and in what circumstances adopting evidence-based core components drove the delivery of an intensive and responsive ESD service, from the perspective of staff members. Findings shed light into how core components interacted with contextual conditions and staff’s responses to lead to intended and unintended outcomes. Despite variations in the wider service model of operation and geographical location, the adoption of core components of ESD such as eligibility criteria and MDT working, helped teams manage the pressures and deliver services that met evidence-based standards, thus, supporting our first programme theory.

The time staff members spent travelling was identified as a key contextual factor, putting a strain on the capacity and MDT working of services covering rural and/or large catchment areas. In line with the second programme theory, the study uncovered differences in how staff in rural services implemented core components compared to urban sites, although services covering large catchment areas were also affected. Future studies may need to distinguish between remoteness and rurality in order to disentangle the influence of geography in delivering an effective service [32].

To protect evidence-based standards and meet their intensity and responsiveness targets, rural teams developed compensatory mechanisms, such as prolonging the service or adjusting MDT processes. Previous research in England and other countries identified low staffing levels as a key factor impacting on the function of rural rehabilitation services and their ability to provide evidence-based care [33, 34]. Though most teams in the current study reported experiencing capacity issues, services covering rural and/or large catchment areas reported to be more sensitive to capacity fluctuations which in turn affected the success of their compensatory mechanisms and their efforts to address contextual challenges. Our findings suggest that appropriate staffing levels for the smooth operation of services needs to be highlighted at a commissioning level and taken into account in developing business cases for service improvement. This fits with recent national policy recommendations in England regarding the implementation of the National Integrated Community Stroke Service model [35].

With regards to team composition, staff accounts permitted a better understanding of the role of RAs in the MDT, complementing our previously published international ESD consensus document on the recommended members of the multidisciplinary team [10, 14]. The need to empower and promote the autonomy of support staff through appropriate supervision and upskilling was stressed. Based on our findings to date, RAs should be recognised as core members of multidisciplinary teams delivering ESD. Respondents also noted the contribution of social care workers in streamlining the discharge process and promoting ESD responsiveness. The boundary spanning function of their work [36] facilitated the timely response of ESD teams and permitted the allocation of targeted slots. The importance of partnership working with social care has been a consistent finding in the ESD literature [19, 37–39] but the issue remains pertinent.

Participants’ accounts reiterated the need to protect MDT meetings, but also allowed us to surface important elements of effective teamwork, including the value of investing in the development of an interdisciplinary skillset. Interdisciplinary working has been previously seen as a way to support the efficiency of activities in the community as it reduces the number of staff required at home visits [40]. Our understanding of the role of interdisciplinary working as a mechanism driving the intensity of rehabilitation provision is in line the concept of ‘role blurring’ in the literature [40]. In addition to supporting staff’s professional development, encouraging skills transfer and cross-boundary working in the MDT, may also directly benefit patients by promoting the continuity of care [40].

Providing a targeted service to eligible patients helped teams protect capacity and deliver an intensive and responsive service. Stretching the criteria to admit more complex patients was common and reflected staff’s attempt to respond to the fragmentation and inflexibility of the care pathways and give more patients the opportunity to benefit from community rehabilitation. In line with previous studies, [10, 19] our findings point to a well-recognised gap in service provision in England for stroke survivors who do not meet the ESD criteria and emphasise the need for a more integrated and comprehensive stroke service provision. Current policy in England supports the implementation of integrated community stroke service models that deliver ESD, but that are also resourced to provide wider community stroke rehabilitation that addresses the needs of patients with more severe disability, providing a more streamlined experience for stroke survivors [12, 35].

This study does not provide an exhaustive account of ESD core components but focuses on those identified by participants as key to the delivery of an intensive and responsive service. A more detailed analysis of staff interviews is provided elsewhere [22]. Our findings are in line with the quantitative investigation conducted as part of the wider WISE study; adopting defined core components of ESD was associated with the provision of a more responsive and intensive ESD service [14]. The qualitative approach of this study allowed us to move beyond the assumption of a causal relationship between mechanisms and outcomes to account for the influence of context and staff’s reasoning. This resonates with current implementation theory, which acknowledges the importance of actors involved in implementation as well as the context in which they are operating [41].

In line with Damschroder’s [13] implementation framework, findings reiterate the importance of distinguishing between core intervention components and an adaptable periphery. For instance, services safeguarded their MDT meetings but adjusted the way that their MDT was organised, to reduce travel time and use resources efficiently. Distinguishing between components that need to be protected for the intervention to be effective and elements that can be modified to account for contextual influences, assists customisation efforts aimed at maximising success of quality improvement initiatives.

To the best of our knowledge, this was the first study to use a RE approach to investigate ESD, building on research investigating interprofessional teamwork in stroke care [42]. Realist evaluation allowed us to address the complexity of ESD delivery in clinical practice and examine how and in what circumstances adopting core components resulted in effective ESD. Rather than seeking to identify every contextual factor at play, the realist approach offered a framework for considering the role of context in relation to the intervention components and social processes that impact outcomes, affording a better understanding of conditional causality (i.e. what works for whom in what circumstances) [43]. The theory-informed approach and the application of the CMO heuristic facilitated the analysis of a very large and information-rich data set. Disaggregating mechanisms between intervention resources and stakeholder reasoning allowed a clearer delineation of mechanisms and contexts. In addition, a pragmatic position with regards to configuring CMOs enabled us to draw take-home messages relevant to clinical practice.

To achieve rigour in developing and reporting the study, the RE quality standards were applied [17, 28]. The analytical task of linking CMO chains is suggested to increase the explanatory power of findings and improve the external validity of case studies [21]. Data were independently analysed by two researchers, reducing ambiguity and enhancing the trustworthiness of our findings. The very process of realist interviewing requires researchers to directly test their hypotheses and assumptions with their participants. Trustworthiness in this study was further established by discussing our findings with participating teams over two feedback workshops. Participants had the opportunity to comment on our interpretations and assess the relevance of our key take home messages with their local settings. It must be acknowledged that data from a relatively small sample of ESD services were used in this study; further research would be required to confirm wider transferability, particularly beyond England. Although it is accepted that the context-specific nature of the findings in realist and case study research limits their quantitative generalisability, the theory-driven nature of the enquiry enhances the transferability of the refined programme theories to other settings with similar characteristics. The cumulation of insights from further testing of these programme theories in different geographical and organisational settings will help develop an evidence-informed theory base for the large-scale implementation of rehabilitation services in the community.

## Conclusions

Overall findings supported the need for adoption of core evidence-based components in achieving a responsive and intensive ESD service. These included the delivery of the service to eligible patients, by well-staffed and co-ordinated multidisciplinary teams. Building on quantitative research findings, this qualitative study helped uncover the processes through which these evidence-based components lead to desirable outcomes in different settings. The study also identified contextual factors that may impede efforts to deliver evidence-based care and services’ attempts to address these barriers by adapting peripheral elements of the intervention. The RE approach allowed us to address the complexities of ESD implementation in clinical settings enabling transferrable lessons to be drawn to inform service improvement efforts. More research is required to develop and test targeted interventions aimed at managing logistical challenges associated with delivering effective home-based stroke rehabilitation in different geographical contexts. Findings highlighted the interdependency between ESD services and the local stroke care pathways and call for a more integrated and comprehensive stroke service provision, responsive to the complex needs of stroke survivors with severe disabilities.

## Data Availability

Due to the qualitative nature of the data we are unable to share them with other researchers for anonymity and confidentiality reasons and in line with our agreements with NHS HRA and Hospital R&D Departments.

## Abbreviations

WISE: What is the Impact of Stroke Early supported discharge?
ESD: Early Supported Discharge
SSNAP: Sentinel Stroke National Audit Programme
RE: Realist Evaluation
CMOs: Context-Mechanisms-Outcomes
MDT: Multidisciplinary team
RA: Rehabilitation assistant
OT: Occupational Therapist
PT: Physiotherapist
GP: General practitioner
